# Synthesis and Application of SAPO-11 Molecular Sieves Prepared from Reaction Gels with Various Templates in the Hydroisomerization of Hexadecane

**DOI:** 10.3390/gels10120792

**Published:** 2024-12-04

**Authors:** Dmitry V. Serebrennikov, Arthur R. Zabirov, Alexey N. Saliev, Roman E. Yakovenko, Tatyana R. Prosochkina, Zulfiya R. Fayzullina, Vladimir Yu. Guskov, Boris I. Kutepov, Marat R. Agliullin

**Affiliations:** 1 Institute of Petrochemistry and Catalysis UFRC RAS, 450075 Ufa, Russia; 2 Research Institute “Nanotechnologies and New Materials”, Platov South-Russian State Polytechnic University, 346428 Novocherkassk, Russia; 3 Faculty of Technology, Ufa State Petroleum Technological University, 450062 Ufa, Russia; 4 Department of Analytical Chemistry, Ufa University of Science and Technology, 450076 Ufa, Russia

**Keywords:** zeolites, molecular sieves, reaction gels, silicoaluminophosphate SAPO-11, nanoscale crystals

## Abstract

Among the most selective catalytic systems for the hydroisomerization of C_16+_ *n*-paraffins, catalytic systems based on SAPO-11 are quite promising. In order to increase the activity and selectivity of these bifunctional catalysts, it is necessary to reduce the diffusion restrictions for the reacting molecules and their products in the microporous structure of SAPO-11 by reducing the crystal size. To solve this problem, we have studied the influence of different templates (diethylamine, dipropylamine, diisopropylamine, and dibutylamine) on the physicochemical properties of reaction gels and SAPO-11 silicoaluminophosphates during their crystallization. Using XRD, SEM, and NMR techniques, we found that regardless of the template used, the reaction gel after the aging process at 90 °C is an AlPO_4_·2H_2_O hydroaluminophosphate. At the same time, the nature of the template affects the morphology and crystal sizes of the intermediate alumophosphate, AlPO_4_·2H_2_O, and the molecular sieves, SAPO-11. The acidic properties and the porous structure characteristics of SAPO-11 are also affected by the template. A template was proposed to enable the synthesis of nanoscale SAPO-11 crystals. The influence of the morphology and crystal size of SAPO-11 on the catalytic properties of a bifunctional catalyst based on SAPO-11 in the hydroisomerization of hexadecane was investigated.

## 1. Introduction

The modern chemical industry’s achievements are closely linked to the use of adsorbents and catalysts based on various types of zeolites, also known as molecular sieves [1,2]. In general, most of the research on molecular sieves and their applications in industrial processes is related to aluminosilicates. However, over the past two decades, there has been an increasing interest in silicoaluminophosphate molecular sieves, known as SAPO-n, which have become of significant interest in industrial processes [3].

SAPO-n molecular sieves were first synthesized by Wilson S.T. and colleagues at Union Carbide in 1984 [4]. Like zeolites, these materials have a wide range of pore sizes and channel structures. For SAPO, the pore size can range from 3.8 Å (SAPO-18) to 12.7 Å (VPI-5). The channels can be one-dimensional (1D, SAPO-11), two-dimensional (2D, SAPO-40), or three-dimensional (3D, SAPO-37) [5].

Acidic sites in SAPO-n are formed by the introduction of silicon into the alumophosphate lattice. Currently, two mechanisms of silicon embedding have been proven: single embedding (SM2), where one silicon atom forms an acidic site, and insular embedding (SM2 + SM3), where silicon is embedded in the form of silicate islands of various sizes [3,6]. In the case of the SM2 + SM3 mechanism, acidic sites are formed at the interface between the SiO_2_ and AlPO_4_ phases. The main difference between SAPO-n and zeolites is the presence of weaker Brønsted acid sites, which allows for higher selectivity in certain catalytic reactions [7,8,9].

Among the various SAPO molecular sieves, SAPO-11 (structural type AEL) is of particular interest. These are characterized by a one-dimensional channel structure with elliptical pores of 4.0 × 6.5 Å and acid sites of moderate strength. Due to this feature, bifunctional catalysts based on these materials are the most selective for the hydroisomerization of higher *n*-paraffins C_7+_ [10].

One of the main challenges preventing an increase in the selectivity of catalysts based on SAPO-11 molecular sieves is the slow diffusion rates of reactant molecules in the microporous structure, which leads to an increase in the residence time of hydroisomerization intermediates in the channels of the molecular sieve. This results in a decrease in the selectivity and stability of the catalyst due to the increased occurrence of side reactions such as the hydrocracking of feed molecules [11]. In order to overcome the diffusion limitations, methods for the synthesis of nanoscale and hierarchical molecular sieves, SAPO-11, have been developed [12,13,14,15,16]. At the same time, the influence of the nature of the template on the morphology and size of their crystals has not been sufficiently studied [17,18]. It is important to note that these parameters are key factors on which the activity and selectivity of catalytic systems based on them depend.

According to modern concepts, during the crystallization of molecular sieves, a template or structure directing agent (SDA) contributes to the formation of porous spaces in the crystal lattices and stabilizes their charges [19]. In addition, by changing the concentration of the template, the pH of the reaction mixture can be adjusted, which is essential for the formation of specific structures. Even in [20], when studying the synthesis of the AlPO_4_-11 molecular sieve using such amines as diethylamine, dipropylamine, diisobutylamine, dibutylamine, butylamine, and hexylamine as templates, it was concluded that for the successful synthesis of the above-mentioned molecular sieve, a secondary amine is needed to create the required electronic effect. In addition, the size of the secondary amine molecules must correspond to a distance along the axis of the molecular sieve unit cell, which is approximately 8.4 Å. Of the templates listed above, only dipropylamine and diisobutylamine meet these criteria and allow for the synthesis of AlPO_4_-11 with high phase purity and high yield.

In the crystallization of SAPO-11 using diethylamine, dipropylamine, diisopropylamine, and a mixture of diethylamine and diisopropylamine as templates, it was found that only dipropylamine and the mixture of diethylamine with diisopropylamine provide the desired material with high phase purity [18,21,22]. In the case of diisopropylamine or diethylamine, SAPO-11 molecular sieves may crystallize with the impurities of SAPO-5 or non-porous aluminophosphates.

In a later study [21], using similar techniques, it was possible to successfully synthesize SAPO-11 with high phase purity using only diethylamine or dipropylamine as templates. The samples obtained with dipropylamine exhibited a higher degree of crystallinity than those obtained with diethylamine. As the authors point out, diisopropylamine is not a selective template, as the crystallization product in this case contains both SAPO-11 and SAPO-5 molecular sieves.

Thus, the literature provides information on the effect of the template on the phase purity of AlPO_4_-11 and SAPO-11 molecular sieves, but it does not practically address the issues related to the influence of the template on the properties of the formed reaction gels, the morphology, and the size of SAPO-11 crystals. As already mentioned, these are key parameters that largely determine the catalytic properties of materials based on SAPO-11 in the hydroisomerization of higher *n*-paraffins C_7+_. This work is dedicated to the study of these issues.

## 2. Results and Discussion

The properties of various molecular sieves are already determined at the stage of the preparation of the reaction mixture, which in the case of silicoaluminophosphates is commonly referred to as the reaction gel [8,23]. Therefore, it is essential to study the physicochemical properties of these reaction gels in order to understand their impact on the properties of the crystallization products.

**Figure 1:**
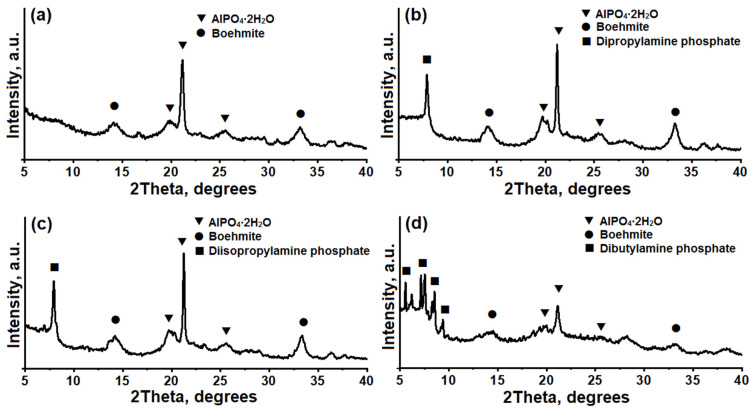
XRD patterns of silicoaluminophosphate gels prepared using various templates: (**a**) Gel-DEA sample, (**b**) Gel-DPA sample, (**c**) Gel-DIPA sample, (**d**) Gel-DBA sample.

Figure 1 shows X-ray diffraction (XRD) patterns of reaction gels prepared using various templates (secondary amines). The gel sample prepared with diethylamine is a mixture of AlPO_4_·2H_2_O hydroaluminophosphate (PDF № 01-070-1391) and unreacted boehmite (PDF № 01-073-9093). When diisopropylamine or dipropylamine was used to prepare the gels, three phases were observed in their compositions: AlPO_4_·2H_2_O, unreacted boehmite, and dipropylamine (PDF № 00-046-0651) phosphate phases. The gel prepared with dibutylamine contains phases of hydroaluminophosphate (AlPO_4_∙2H_2_O) and unreacted boehmite, as well as a phase of dibutylamine phosphate (identified by analogy with the dipropylamine phosphate phase). Therefore, based on the results obtained, it can be concluded that the prepared gels are multiphase colloidal systems whose composition may depend on the template used for their preparation.

**Figure 2:**
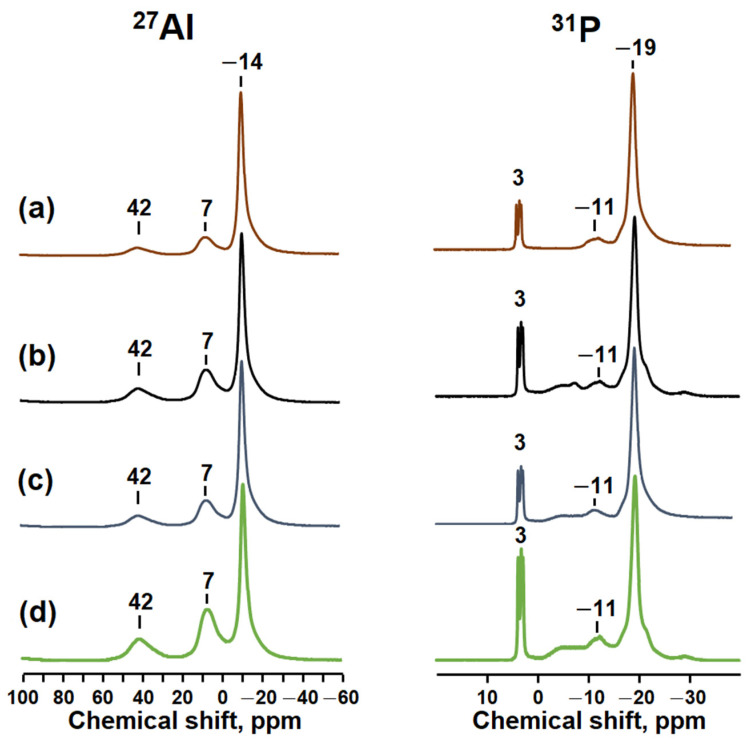
^27^Al and ^31^P MAS NMR spectra of silicoaluminophosphate gels prepared using various templates: (**a**) Gel-DEA sample, (**b**) Gel-DPA sample, (**c**) Gel-DIPA sample, (**d**) Gel-DBA sample.

To better understand the effect of the secondary amine on the interaction between aluminum and the phosphorus source, we recorded ^27^Al and ^31^P MAS NMR spectra for the formed reaction gels, which are shown in Figure 2. All ^27^Al spectra show three main signals at 7, −14, and 42 parts per million (ppm). The signal at 7 ppm is typically attributed to aluminum atoms in an octahedral oxygen environment [AlO_6_], which is characteristic of boehmite [8,24,25,26,27].

A strong signal at −14 ppm is attributed to aluminum atoms in an oxygen environment [AlO_6_] within crystalline alumophosphates, AlPO_4_∙2H_2_O. A weaker signal at 42 ppm is also present, attributed to aluminum atoms in a tetrahedral environment [AlO_4_] in amorphous aluminophosphates [8,24,25,26,27]. Comparing the spectra of the reaction gels, we can see that there is an increase in signal intensity at 7 ppm in the spectrum of dibutylamine compared to the spectrum of diethylamine. This indicates a higher proportion of unreacted boehmite in the gel composition for dibutylamine.

From these results it can be concluded that there is a weaker interaction between the aluminum and phosphorus sources in the gels as the molecular weight of the template increases.

In the ^31^P NMR spectra of all the gels, there is an intense signal at −19 ppm, which is usually associated with phosphorus atoms in a tetrahedral oxygen environment [PO_4_], as found in crystalline aluminophosphates. In this case, it is present as AlPO_4_∙2H_2_O [8,24,25,26,27]. There are also weak signals at −11 and 3 ppm. The signal at −11 ppm is attributed to phosphorus atoms in alumophosphates of the type P(OAl)_n_OH (n varies from 1 to 4), and the signal at 3 ppm is attributed to phosphorus atoms in aminophosphates [8,24,25,26,27]. A comparison of the two spectra shows that in the spectrum of a gel prepared using diethylamine, the signal at −19 ppm is slightly more intense and the signal at 3 ppm is reduced compared to the spectrum of a gel obtained using dibutylamine.

The results obtained allow us to conclude that the degree of interaction between aluminum and phosphorous sources decreases as the molecular weight of the template increases and are in good agreement with the pH values of the reaction mixtures. It can be observed that during the transition from diethylamine to dibutylamine, there is a small decrease in the pH value, indicating a decrease in the amount of aluminophosphate in the reaction gels.

**Figure 3:**
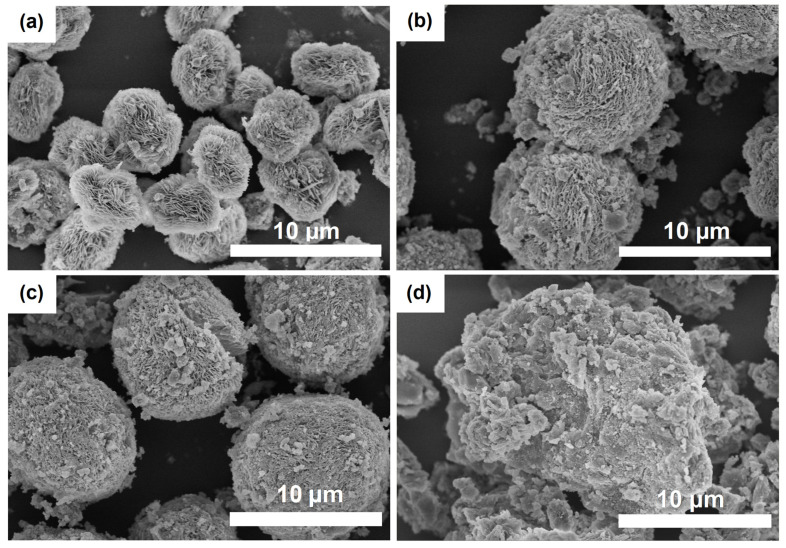
SEM images of silicoaluminophosphate gels prepared using various templates: (**a**) Gel-DEA sample, (**b**) Gel-DPA sample, (**c**) Gel-DIPA sample, (**d**) Gel-DBA sample.

Figure 3 shows scanning electron microscopy (SEM) images of gel samples prepared using different templates. As seen, the dried gel samples are spherical aggregates of different sizes, composed of thin plates. From the XRD data, it can be concluded that these aggregates are crystalline hydroaluminophosphates, AlPO_4_∙2H_2_O. In addition, as the molecular weight of the amine increases, the size of the spherical aggregates also increases. Therefore, it seems that the nature of the secondary template plays a significant role in determining the depth of reaction between the aluminum and phosphorus sources in the gel mixture, as well as influencing the phase composition, morphology, and size of the crystals present in the final product.

It is known that as the molecular weight of the hydrocarbon substituent in secondary amines increases, their basicity also increases. This is probably due to the stronger interaction between high basicity amines and phosphoric acid, leading to the formation of amine phosphates that are less reactive in subsequent interactions with aluminum sources. Previously, it was shown in [24] that for the synthesis of aluminophosphate molecular sieves, it is essential to ensure a stronger interaction between the aluminum and phosphorus sources leading to the formation of ≡Al-O-P≡ bonds and aluminophosphate formation.

**Figure 4:**
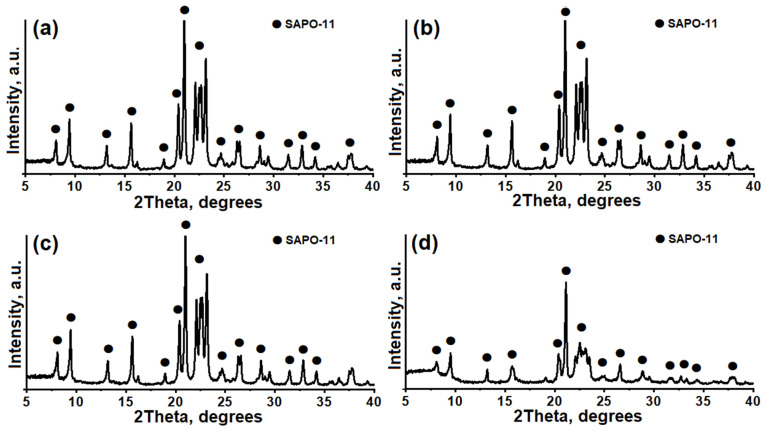
XRD patterns of SAPO-11 molecular sieves prepared using various templates: (**a**) SAPO-11 (DEA) sample, (**b**) SAPO-11 (DPA) sample, (**c**) SAPO-11 (DIPA) sample, (**d**) SAPO-11 (DBA) sample.

The XRD patterns of the crystallization products of silicoaluminophosphate gels prepared using various templates are shown in Figure 4. Table 1 provides information on their phase composition and crystallinity. As can be seen, regardless of the template used, the main crystallized product in all gels is silicoaluminophosphate, also known as the SAPO-11 molecular sieve (PDF № 00-041-0023), for which the following characteristic peaks are defined: 2Θ = 8.11, 9.47, 13.20, 15.68, 20.41, 21.06, 22.13, 22.52, 22.73, 23.20.

Samples of SAPO-11 prepared with diethylamine, dipropylamine, and diisopropylamine are characterized by higher crystallinity compared to the sample prepared with dibutylamine.

The results obtained contradict those of [20], which suggested that a secondary amine is required for the successful synthesis of molecular sieves with the AEL structure. The size of these molecules must correspond to a distance along the c-axis of the molecular sieve unit cell equal to approximately 8.4 Å. Only dipropylamine and diisopropylamine meet these requirements. Our results indicate that the synthesis of SAPO-11 molecular sieves is feasible with all the studied secondary amines. Apparently, the phase purity of molecular sieves with the AEL structure depends not only on the nature of the template but also on the source of silicon, its concentration, and the conditions of gel aging during the reaction.

**Figure 5:**
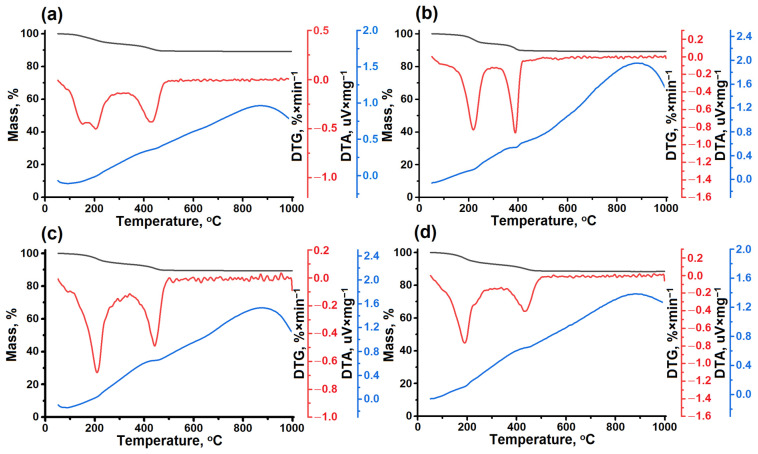
TG/DTG/DTA curves of noncalcined SAPO-11 molecular sieves prepared using various templates: (**a**) SAPO-11 (DEA) sample, (**b**) SAPO-11 (DPA) sample, (**c**) SAPO-11 (DIPA) sample, (**d**) SAPO-11 (DBA) sample.

Thermogravimetry was used to determine the content of various templates in the molecular sieve samples and to determine the temperature of the SAPO-11 heat treatment at which the amine was completely removed. Figure 5 shows the results of the derivatography, while Table 1 presents the calculated content of template molecules per unit cell.

As seen from the TG and DTG curves, three mass loss peaks are observed on the differential curve for all SAPO-11 samples. The first peak in the temperature range of 50 to 100 °C is associated with the desorption of intracellular water, the second peak in the range of 150 to 300 °C is associated with the desorption of physically adsorbed secondary amines, and the third peak in the range of 300 to 400 °C is related to the decomposition of secondary amines at the Brønsted acid sites (Hoffman decomposition).

Two endothermic peaks were observed on the DTA curves for all samples. The first peak, between 150 and 300 °C, is associated with the desorption of physically adsorbed templates. The second peak, between 300 and 400 °C, corresponds to the decomposition of the templates into BASs.

As seen, as the molecular weight of the amine increases, the SDA/unit cell ratio in the SAPO-11 molecular sieve decreases. The highest SDA/unit cell value was found in the SAPO-11 (DEA) sample. The SDA/unit cell ratio of the SAPO-11 (DIPA) sample is slightly lower than that of the SAPO-11 (DPA) sample. The lowest SDA/unit cell value is typical for the SAPO-11 (DBA) sample.

**Figure 6:**
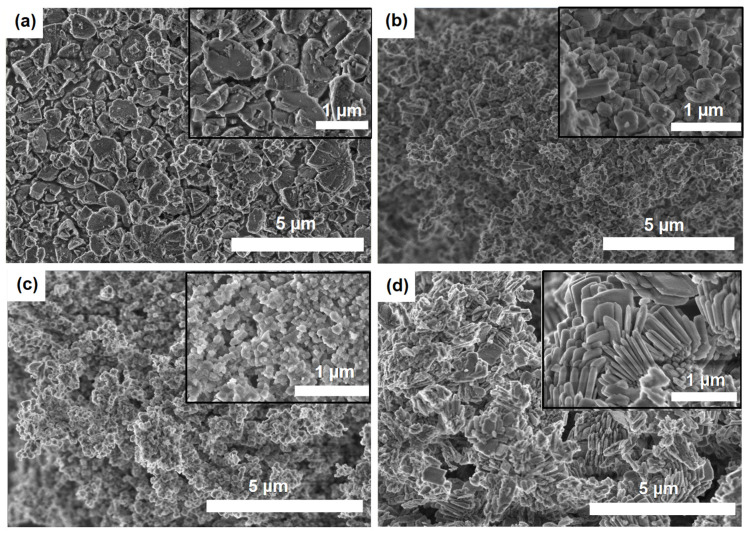
SEM images of SAPO-11 molecular sieves prepared using various templates: (**a**) SAPO-11 (DEA) sample, (**b**) SAPO-11 (DPA) sample, (**c**) SAPO-11 (DIPA) sample, (**d**) SAPO-11 (DBA) sample.

It is known that the activity and selectivity of catalytic systems based on molecular sieves depend strongly on their morphology and crystal size of the latter [11]. Scanning electron microscope (SEM) images of molecular sieves synthesized using different templates are shown in Figure 6.

As seen, the morphology and crystal size of SAPO-11 molecular sieves are influenced by the nature of the template. The SAPO-11 (DEA) sample has cone-shaped crystals with a size of approximately 1000 nm (Figure 6a). The SAPO-11 (DPA) sample has nanoscale crystals in the form of truncated cones with a size range between 150 and 250 nm (Figure 6b).

The use of diisopropylamine as a template allows for the preparation of SAPO-11 (DIPA) crystals with an even smaller size, ranging from 100 to 200 nm (Figure 6c), and a cubic morphology. In contrast, the use of dibutylamine as a template leads to the formation of SAPO-11 (DBA) (Figure 6d), which takes the form of thin plates with dimensions ranging from 500 to 1000 nm in length and between 100 and 200 nm thick.

**Figure 7:**
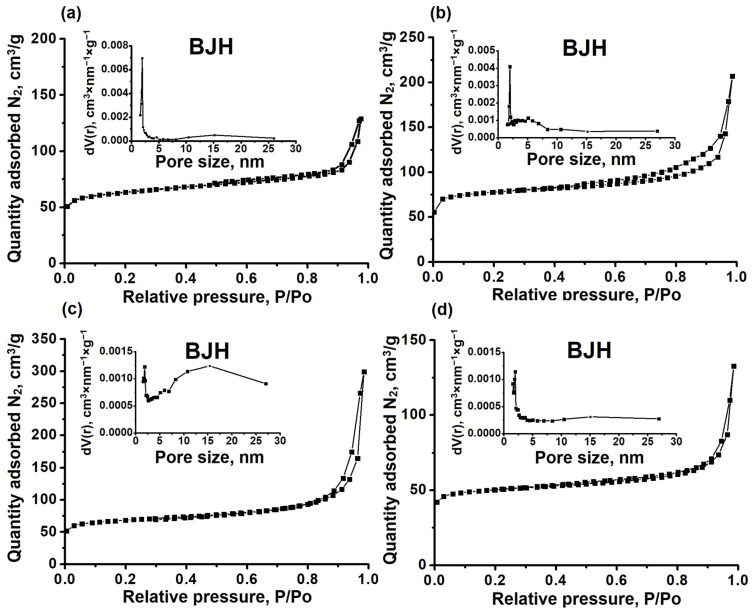
Nitrogen adsorption–desorption isotherms and pore size distribution of SAPO-11 molecular sieves prepared using various templates: (**a**) SAPO-11 (DEA) sample, (**b**) SAPO-11 (DPA) sample, (**c**) SAPO-11 (DIPA) sample, (**d**) SAPO-11 (DBA) sample.

Figure 7 demonstrates the nitrogen adsorption–desorption isotherms and pore size distribution, and Table 2 shows the porous structure characteristics of SAPO-11 molecular sieves synthesized using various templates. The SAPO-11 (DEA) sample has a type I isotherm as shown in Figure 7a. This type of isotherm is typical for microporous materials that have almost no secondary meso- or macropores. SAPO-11 (DPA) (Figure 7b), SAPO-11 (DIPA) (Figure 7c), and SAPO-11 (DBA) (Figure 7d) show isotherms close to type IV, with a sharp increase in the low-pressure region and an H3-type hysteresis loop in the higher pressure region. This isotherm type is characteristic of micro-mesoporous materials.

The formation of mesopores in these samples is due to the incomplete fusion of nanoscale crystals, as can be seen from the SEM images in Figure 6. Mesopores in SAPO-11 (DBA) form as a result of the fusion of thin platelets. SAPO-11 (DPA) and SAPO-11 (DIPA) samples have the largest mesopore volume, allowing these molecular sieves to be classified as hierarchical porous materials.

The highest specific surface area and the largest volume of mesopores in the SAPO-11 (DIPA) sample are due to the formation of a secondary porous structure by the smallest crystals (100–200 nm in size).

**Figure 8:**
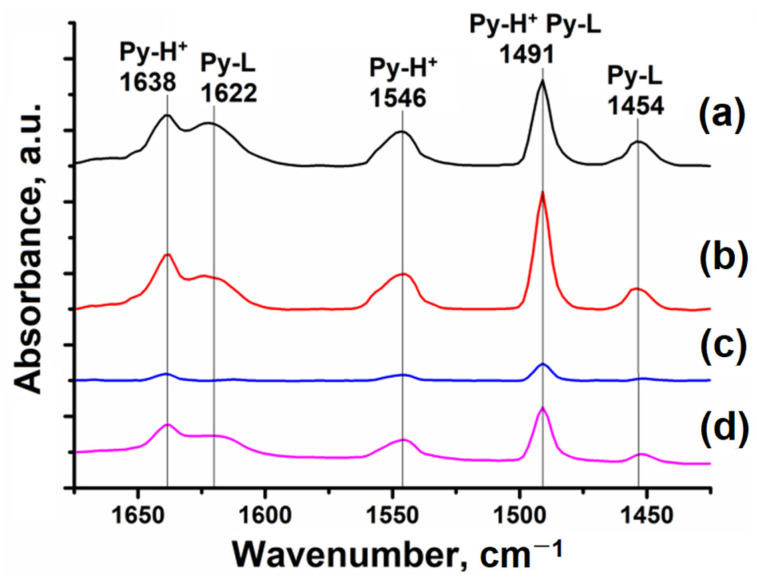
IR spectra of adsorbed pyridine for SAPO-11 after desorption at 150 °C: (**a**) SAPO-11 (DIPA) sample; (**b**) SAPO-11 (DPA) sample; (**c**) SAPO-11 (DBA) sample; (**d**) SAPO-11 (DEA) sample.

The obtained SAPO-11 samples were analyzed using pyridine adsorption/desorption combined with IR spectroscopy (Figure 8). Several pyridine bands were observed in the IR spectra, as shown in Figure 8: 1638 cm^−1^ and 1546 cm^−1^ are associated with pyridine adsorbed on Brønsted acid sites (BASs); 1622 cm^−1^ and 1454 cm^−1^ correspond to pyridine adsorbed on Lewis acid sites (LASs); and 1491 cm^−1^ is associated with BASs and LASs.

Nanocrystalline SAPO-11 (DIPA) and SAPO-11 (DPA) are characterized by having the highest concentrations of BASs and LASs (Table 3). The use of DEA as a template resulted in the formation of larger SAPO-11 crystals during synthesis, which had less accessible acid sites, resulting in a lower concentration of these sites on pyridine adsorption compared to samples obtained using DIPA and DPA. SAPO-11, synthesized using DBA, is characterized by a low concentration of acid sites, which is attributed to its low silicon content and the presence of large crystals that make some of the acid sites inaccessible to the pyridine probe molecule (Table 1).

Pt/SAPO-11 samples were prepared by impregnation with an aqueous solution of H_2_PtCl_6_ at 0.5 wt.% Pt per sample, followed by drying and calcination. As shown previously [28], this method of preparing Pt-containing SAPO-11 samples allows for a high dispersion of the specified concentration. Therefore, the limiting stage in the hydroisomerization process is mainly the transformations at acid sites.

It has been found that the hydroisomerization of hexadecane over Pt/SAPO-11 under these conditions at up to 300 °C (conversion up to 80–90%) produced mainly monobranched isomers of hexadecane (2-, 3-, 4-, 5-, 6-, 7-, and 8-methylpentadecanes) as well as a certain amount of various dibranched and more branched isomers of hexadecane and lower molecular hydrocarbons, which are their destruction products. As the reaction temperature is further increased, the content of branched isomers increases and, at the same time, the products of their destruction are observed.

**Figure 9:**
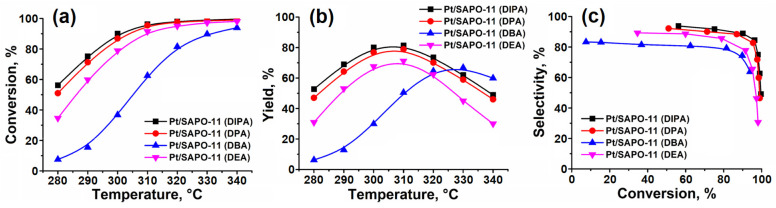
Hydroisomerization of hexadecane over 0.5 wt.% Pt/SAPO-11 samples: (**a**) hexadecane conversion versus reaction temperature; (**b**) C_16_ isomer yield versus reaction temperature; (**c**) C_16_ isomer selectivity versus hexadecane conversion.

Figure 9 shows that the Pt/SAPO-11 (DIPA) and Pt/SAPO-11 (DPA) samples, prepared using diisopropylamine and dipropylamine as templates, have high activity in the hydroisomerization of hexadecane. The conversion of C_16_ at 310 °C was 95–96%, with yields of C_16_ isomers of 81% and 79% for the DIPA and DPA samples, respectively.

These SAPO-11 samples are characterized by their nanoscale crystals (Figure 6) and the highest concentration of acid sites (Table 3). Pt-containing samples based on larger SAPO-11 crystals (DEA and DBA) have a lower concentration of these active sites and are therefore less active in the hydroisomerization reaction. These samples achieve a degree of C_16_ conversion at 310 °C of 91% for DEA and 63% for DBA.

The selectivity for C_16_ isomers decreases with increasing crystal size, while the concentration of acid sites decreases in the SAPO-11 series: Pt/SAPO-11 (DIPA) > Pt/SAPO-11 (DPA) > Pt/SAPO-11 (DEA) > Pt/SAPO-11 (DBA).

## 3. Conclusions

The influence of the properties of silicoaluminophosphate (SAPO) reaction gels prepared using different templates (diethylamine, dipropylamine, diisopropylamine, and dibutylamine) on the crystallization of SAPO-11 molecular sieves was investigated.

The template (SDA) significantly influences the phase composition of the reaction gels that are formed. The reaction gels prepared using different templates consist of a mixture of boehmite, amine phosphate, and alumophosphate (AlPO_4_∙2H_2_O), with varying proportions of each component.

As the molecular weight of SDA increases, the depth of interaction between the aluminum and phosphorus sources decreases. The structure of the reaction gel is composed of spherical aggregates of different sizes, mainly consisting of thin plates of AlPO_4_∙2H_2_O. An increase in the molecular weight of the amine leads to an increase in the size of these spherical aggregates.

The crystallization of gels with the composition 1.0Al_2_O_3_∙1.0P_2_O_5_∙0.3SiO_2_∙1.0SDA∙40H_2_O, regardless of the template used, leads to the formation of SAPO-11 molecular sieves with high phase purity. As the molecular weight of the amine increases, the SDA/unit cell molar ratio in the SAPO-11 molecular sieve decreases, as does the silicon content in the crystal lattice. The highest value of the SDA/unit cell is observed in the SAPO-11 (DEA) sample.

The influence of the template on the crystal size, morphology, and properties of the secondary porous structure of SAPO-11 was determined. It was found that using dipropylamine and diisopropylamine as an SDA allows for the preparation of nanocrystalline SAPO-11 with a hierarchical porous structure.

The smaller crystal size of SAPO-11 provides improved accessibility of acid sites, reduced diffusion limitations, and reduced residence time of reaction products within the channels compared to larger crystal samples synthesized with diethylamine or dibutylamine. As a result, higher hexadecane conversion and higher selectivity for C_16_ isomers are achieved over the synthesized nanocrystalline SAPO-11. The results obtained suggest that it is possible to control the morphology and size of the crystals in SAPO-11 molecular sieves and further to create bifunctional catalytic systems with different activities and selectivities for the hydroisomerization of higher *n*-paraffins C_16+_ simply by changing the template.

## 4. Materials and Methods

### 4.1. Preparation of Silicoaluminophosphate Gels

Silicoaluminophosphate reaction gels with the following composition were prepared for the synthesis of SAPO-11 molecular sieves: 1.0Al_2_O_3_∙1.0P_2_O_5_∙0.3SiO_2_∙1.0SDA∙40H_2_O, where SDA is the template. For the preparation, boehmite (PB, AlO(OH), 78% Al_2_O_3_, Sasol SB, Hamburg, Germany) and orthophosphoric acid (85%, H_3_PO_4_, Reachem, Moscow, Russia) were used as sources of aluminum and phosphorus, respectively. Diethylamine (DEA, 99%, Sigma-Aldrich, Darmstadt, Germany) and dipropylamine (DPA, 99%, Acros Organics, Schwerte, Germany), diisopropylamine (DIPA, 99%, Sigma-Aldrich, Darmstadt, Germany), and dibutylamine (DBA, 99%, Sigma-Aldrich, Darmstadt, Germany) were also used as templates.

Reaction gels were prepared by adding 10.0 g of orthophosphoric acid to 27.0 g of distilled water followed by the addition of 5.6 g of boehmite with intensive stirring. Subsequently, the calculated amount of template was introduced to the gel, dependent on the composition of the reaction mixture. The following quantities of the respective compounds were added: DEA (3.2 g), DPA (4.4 g), DIPA (4.4 g), and DBA (5.6 g).

The prepared gels were exposed to 90 °C for 24 h, as shown in [24], in order to avoid the formation of non-porous tridymite during subsequent crystallization. The gels prepared using different templates, namely DEA, DPA, DIPA, and DBA, are designated as Gel-DEA, Gel-DPA, Gel-DIPA, and Gel-DBA, respectively.

### 4.2. Crystallization of SAPO-11 Molecular Sieves

SAPO-11 molecular sieves were synthesized via hydrothermal crystallization of reaction gels at 200 °C for 24 h. SAPO-11 samples obtained using DEA, DPA, DIPA, and DBA are designated as SAPO-11 (DEA), SAPO-11 (DPA), SAPO-11 (DIPA), and SAPO-11 (DBA), respectively.

### 4.3. Preparation of Bifunctional Catalytic Systems

Pt-containing catalysts based on the SAPO-11 molecular sieve were prepared as follows: a 0.1–0.5 mm fraction was precalcined at 600 °C for 6 h in an air atmosphere and then impregnated with an aqueous solution of H_2_PtCl_6_·6H_2_O (99%, Sigma Aldrich, Darmstadt, Germany). The platinum content was 0.5 wt. % of the support weight. The sample was then dried at 100 °C for 24 h and calcined at 550 °C for 5 h. Before starting the reaction, the catalyst was reduced in a hydrogen flow (100 mL/min) at 400 °C for 5 h. The conditions for activation of the catalyst samples were chosen according to the references [10,14].

### 4.4. Methods of Materials Analysis

X-ray fluorescence spectroscopy on a Shimadzu EDX-7000P spectrometer (Shimadzu Corporation, Duisburg, Germany) was used to determine the chemical composition of the reaction gels and silicoaluminophosphate molecular sieves.

The phase compositions of reaction gels and SAPO-11 molecular sieve samples were determined using a Shimadzu XRD 7000 diffractometer (Shimadzu Corporation, Kyoto, Japan) in CuKa radiation. The device scanned the angle range of 2θ from 3° to 40°, with a scanning speed of 1° per minute. The X-ray data were processed, and the phases were analyzed using the Shimadzu XRD program (version 7.04) with the PDF2 database (version 2.2201). Crystallinity was evaluated based on the presence of an amorphous halo within the range of 20° to 30° 2θ, as determined by the Shimadzu XRD Cristalinity program (version 7.04).

The coordination environment of aluminum and phosphorus atoms was evaluated using ^27^Al and ^31^P NMR spectra. The spectra were obtained using the Avance—400 Bruker NMR spectrometer (Bruker Corporation, Billerica, MA, USA) under conditions of a single-pulse experiment when samples rotated at a magic angle (~7 kHz) in zirconium dioxide rotors (Ø 4mm). The Larmor frequency is 104 MHz (^27^Al) and 162 MHz (^31^P), with a π/2 pulse duration of 2 µs (^31^P) and 2.5 µs (^27^Al).

Thermogravimetric studies of molecular sieve samples were conducted on a synchronous thermal analyzer, STA 449 F5 (Netzsch, Selb, Germany), under the following conditions: sample weight between 20 and 30 mg and using a corundum crucible. The temperature varied from 50 °C to 1000 °C, with a heating rate of 10 °C/min. An inert medium (helium gas) was used, and the gas flow rate was 60 cm^3^/min.

The morphology and crystal sizes of SAPO-11 samples were examined using scanning electron microscopy (SEM) with field emission on a Hitachi Regulus SU 8220 scanning electron microscope (Hitachi High-Tech Corporation, Tokyo, Japan). Images were captured in secondary electron mode at an acceleration voltage of 5 kV.

The characteristics of the porous structure were obtained using the low-temperature nitrogen adsorption–desorption method on a Quantachrome Nova 1200e sorbtometer (Quantachrome Instruments, Boynton Beach, FL, USA). Specific surface area was obtained using the BET (Brunauer–Emmett–Teller) method. The t-plot method was applied to calculate the volume of micropores, while the BJH (Barret–Joyner–Halenda) model was used to determine the pore size distribution.

The types and concentrations of acid sites were determined by IR spectroscopy with pyridine adsorption. The IR spectra were recorded on a Bruker Vertex-70V FT-IR spectrometer (Bruker Optic GmbH, Ettlingen, Germany) with a resolution of 4 cm^−1^. The molecular sieve samples were precalcined at 450 °C in a vacuum. Pyridine was adsorbed onto samples for 30 min and then desorbed at 150, 250, and 350 °C. Brønsted (BAS) and Lewis (LAS) acid site concentrations were calculated by integrating the absorbance bands at 1545 and 1454 cm^−1^, respectively, using the method described in reference [29].

### 4.5. The Hydroisomerization Reaction of Hexadecane

The hydroisomerization of hexadecane (C_16_H_34_, 99%, Acros Organics, Morris Plains, NJ, USA) was studied in a flow reactor at temperatures between 280 and 340 °C, under an H_2_ pressure of 3.0 MPa, with an H_2_/C_16_H_34_ molar ratio of 10, a C_16_H_34_ flow rate of 0.1 mL/min, and a weight hourly space velocity of 2 h^−1^. The conditions for the catalytic tests were chosen according to the references [10,22,30]. Products of catalytic transformations of hexadecane were analyzed by gas chromatography on a Chromatec-Crystal 5000 chromatograph (Chromatec, Yoshkar-Ola, Russia) with a flame ionization detector and an HP-1 capillary column (50 m × 0.2 mm, 0.5 μm, Agilent, Santa Clara, CA, USA).

## Figures and Tables

**Table 1:** Chemical composition and template content of SAPO-11 molecular sieves.

Sample	Gel	SAPO-11	SDA/Unit Cell
SAPO-11 (DEA)	Al_1.00_P_0.99_Si_0.15_	Al_1.00_P_0.92_Si_0.13_	3.59
SAPO-11 (DPA)	Al_1.00_P_0.98_Si_0.15_	Al_1.00_P_0.93_Si_0.12_	2.47
SAPO-11 (DIPA)	Al_1.00_P_0.99_Si_0.15_	Al_1.00_P_0.91_Si_0.14_	2.64
SAPO-11 (DBA)	Al_1.00_P_0.97_Si_0.15_	Al_1.00_P_0.96_Si_0.06_	1.70

**Table 2:** Characteristics of the porous structure of SAPO-11 molecular sieves prepared using various templates.

Sample	S_BET_, m^2^/g	V_micro_, cm^3^/g	V_meso_, cm^3^/g
SAPO-11 (DEA)	203	0.06	0.11
SAPO-11 (DPA)	245	0.07	0.20
SAPO-11 (DIPA)	251	0.07	0.25
SAPO-11 (DBA)	159	0.05	0.10

Symbol: S_BET_—specific surface according to BET; V_micro_—specific volume of micropores; V_meso_—specific volume of mesopores.

**Table 3:** Concentrations of acid sites of SAPO-11 according to IR spectroscopy data with pyridine adsorption.

Sample	Acidity (μmol/g)					
	BAS			LAS		
	150 °C	250 °C	350 °C	150 °C	250 °C	350 °C
SAPO-11 (DIPA)	136	112	34	40	19	11
SAPO-11 (DPA)	121	103	26	51	26	16
SAPO-11 (DBA)	15	13	2	4	1	1
SAPO-11 (DEA)	74	38	14	14	7	3

## Data Availability

The original contributions presented in the study are included in the article, further inquiries can be directed to the corresponding authors.
